# Supplementary material for: Strategic tradeoffs in competitor dynamics on adaptive networks
Source: Sci Rep. 2017 Aug 8;7:7576. doi: 10.1038/s41598-017-07621-x (PMC5548779; doi:10.1038/s41598-017-07621-x)
Supplement: Supplementary file 1 — Supplementary Methods [file 41598_2017_7621_MOESM1_ESM.pdf]

Strategic tradeoffs in competitor dynamics on adaptive networks [Supplementary Methods]

Laurent Hébert-Dufresne,[1,2] Antoine Allard,[3] Pierre-André Noël,[4] Jean-Gabriel Young,[5] and Eric Libby[1,*]

[1] *Santa Fe Institute, Santa Fe, NM 87501, USA*
[2] *Institute for Disease Modeling, Bellevue, WA, 98005, USA*
[3] *Centre de Recerca Matemàtica, E-08193 Bellaterra (Barcelona), Spain*
[4] *University of California, Davis CA 95616, USA*
[5] *Département de physique, de génie physique et d'optique, Université Laval, Québec (Qc), G1V 0A6, Canada*

This document provides a full analysis of the model described in the main text. We provide general mean-field equations for the dynamics in which an arbitrary number of competitors are at play. We describe all possible outcomes of the case with two competitors, and solve all fixed points of the dynamics in the case with an arbitrary number of competitors. We also reproduce the full data from Ref. [13] in main text, use them to estimate our model parameters and produce the corresponding flow diagrams.

I. GENERAL FORMULATION OF THE MODEL

We consider a population of N nodes belonging at any given time to one of a set of different competitors, noted $\mathcal{G}$. At time t, $N_i(t)$ nodes belong to competitor $i \in \mathcal{G}$ during the interval $[t, t+dt)$. The interactions between the nodes are prescribed by the *directed* stochastic block model specified by the matrix $\boldsymbol{P}$ whose elements p_{ij} correspond to the probability that a directed link exists from a node of state i towards a node of state j. Nodes continuously change hands by randomly switching to the state of one of the nodes that have a directed link pointing to them (i.e., incoming link). Hence, at any time t, a node of state i has $k_{ij}^{\mathrm{in}}(t)$ incoming links from nodes of state j with probability

$$\Pr[k_{ij}^{\mathrm{in}}(t) = n] = \binom{N_j(t)}{n} p_{ji}^n (1-p_{ji})^{N_j(t)-n} \ , \tag{1}$$

and opts for state j with a probability $k_{ij}^{\mathrm{in}}(t)/\sum_{l\in\mathcal{G}} k_{il}^{\mathrm{in}}(t)$. Once a node has changed state, its incoming and outgoing links are redrawn according to the matrix $\boldsymbol{P}$. Note that nodes may stick to their current state since self-loops are allowed (i.e., stubbornness) and incoming links may be from nodes sharing the same state (i.e., group cohesion).

Let us now state the general mean-field equations to follow the dynamics in the limit of an infinite population being disputed by a set $\mathcal{G}$ of different competitors. To do so, we consider an *annealed* version of the model in which links are continuously redrawn according to matrix $\boldsymbol{P}$ regardless of whether nodes change their state or not, and this is achieved at a rate faster than the process of switching states itself (i.e., there is no temporal correlation). We also consider the limit $N \to \infty$ and define $x_i(t) = N_i(t)/N$ as the fraction of nodes sharing state i. Note that we will explicitly write the time dependency only if required to avoid confusion. A node of state i has a number of incoming links from nodes of state j proportional to $p_{ji}x_j$, and switched its state to j at a rate proportional to $p_{ji}x_j/\sum_{l\in\mathcal{G}} p_{li}x_l$. Altogether, the time evolution of the system is described by the following differential equation

$$\dot{x}_i = x_i \sum_j x_j \left(p_{ij}\varphi_j - p_{ji}\varphi_i\right) \qquad \forall\ i \in \mathcal{G} \tag{2}$$

where the elements of the matrix $\boldsymbol{P}$, $\{p_{ij}\}_{i,j\in\mathcal{G}}$, are the probability that a directed link exists from a node of competitor j towards a node of competitor i, and where φ_j is the reciprocal of the expected in-degree of nodes of competitor j

$$\varphi_j = \frac{1}{\sum_l p_{lj} x_l} \ . \tag{3}$$

* Correspondence to : elibby@santafe.edu

We can readily compute the elements of the Jacobian matrix from which the stability of the fixed points of Eq. (2) can be determined

$$J_{ki} = \frac{\partial \dot{x}_k}{\partial x_i} = x_k \sum_j \frac{\partial x_j}{\partial x_i}\big(p_{kj}\varphi_j - p_{jk}\varphi_k\big) + \frac{\partial x_k}{\partial x_i}\sum_j x_j\big(p_{kj}\varphi_j - p_{jk}\varphi_k\big) + x_k \sum_j x_j \left(p_{kj}\frac{\partial \varphi_j}{\partial x_i} - p_{jk}\frac{\partial \varphi_k}{\partial x_i}\right)$$
$$= x_k\big(p_{ki}\varphi_i - p_{ik}\varphi_k\big) + \delta_{ik}\sum_j x_j\big(p_{kj}\varphi_j - p_{jk}\varphi_k\big) + x_k \sum_j x_j\big(p_{jk}p_{ik}\varphi_k^2 - p_{kj}p_{ij}\varphi_j^2\big) \ . \tag{4}$$

Let us finally rewrite Eq. (2) in a way that will be useful in the subsequent analysis.

$$\dot{x}_k = x_k\left(\sum_j p_{kj}x_j\varphi_j - 1\right) \ . \tag{5}$$

II. GENERAL SOLUTION FOR THE CASE $|\mathcal{G}| = 2$

Let us consider the two-competitor scenario, $\mathcal{G} = \{1, 2\}$, first without any constraint on the elements of the matrix $\boldsymbol{P}$. The conservation of nodes, $x_1 + x_2 = 1$, implies that the dynamics of the system, Eq. (2), can be tracked with the single equation

$$\dot{x}_1 = x_1(1 - x_1)\left[\frac{1}{(1-\alpha_2)x_1 + \alpha_2} - \frac{1}{(\alpha_1 - 1)x_1 + 1}\right] \ , \tag{6}$$

where we have defined $\alpha_1 = p_{11}/p_{21}$ and $\alpha_2 = p_{22}/p_{12}$. Note that $\alpha_1, \alpha_2 \geq 0$ since the elements of matrix $\boldsymbol{P}$ are probabilities. Note also that the system is static regardless of its initial conditions whenever $\alpha_1 = \alpha_2 = 1$. From Eq. (6), we can readily identify the three possible fixed points

$$x_1^{*(1)} = 0 \ ; \qquad x_1^{*(2)} = 1 \ ; \qquad x_1^{*(3)} = \frac{1}{1 + \frac{(\alpha_1 - 1)}{(\alpha_2 - 1)}} \ , \tag{7}$$

with $0 \leq x_1^{*(3)} \leq 1$ whenever

$$\frac{(\alpha_1 - 1)}{(\alpha_2 - 1)} \geq 0 \ . \tag{8}$$

From Eq. (4), we see that the elements of the Jacobian matrix become

$$J_{11} = \ \ x_2(\beta_{12} - \beta_{21}) + x_1x_2(\beta_{11}\beta_{21} - \beta_{12}^2) \tag{9a}$$
$$J_{12} = \ \ x_1(\beta_{12} - \beta_{21}) - x_1x_2(\beta_{12}\beta_{22} - \beta_{21}^2) \tag{9b}$$
$$J_{21} = -x_2(\beta_{12} - \beta_{21}) - x_1x_2(\beta_{11}\beta_{21} - \beta_{12}^2) \tag{9c}$$
$$J_{22} = -x_1(\beta_{12} - \beta_{21}) + x_1x_2(\beta_{12}\beta_{22} - \beta_{21}^2) \tag{9d}$$

where we have defined $\beta_{ij} = p_{ij}\varphi_j$. Noting that $J_{11}J_{22} - J_{12}J_{21} = 0$, we find that the eigenvalues, λ, of the Jacobian matrix are the solution of

$$\lambda(J_{11} + J_{22} - \lambda) = 0 \ . \tag{10}$$

The first eigenvalue $\lambda_1 = 0$ is the result of the conservation of the nodes limiting the dynamics to a single line in the phase space (x_1, x_2). The second eigenvalue, λ_2, is

$$\lambda_2 = (x_2 - x_1)(\beta_{12} - \beta_{21}) + x_1x_2(\beta_{11}\beta_{21} + \beta_{12}\beta_{22} - \beta_{12}^2 - \beta_{21}^2) \ . \tag{11}$$

Substituting Eq. (7) into this last result, we find that each fixed point is stable whenever

$$\lambda_2^{(1)} = \frac{1}{\alpha_2} - 1 < 0 \tag{12a}$$
$$\lambda_2^{(2)} = \frac{1}{\alpha_1} - 1 < 0 \tag{12b}$$
$$\lambda_2^{(3)} = x_1^{*(3)}\left(1 - x_1^{*(3)}\right)\beta_{12}^2(\alpha_1 + \alpha_2 - 2) < 0 \ . \tag{12c}$$

Using these results, the condition (8) can be written as

$$\frac{\alpha_1 \lambda_2^{(2)}}{\alpha_2 \lambda_2^{(1)}} \geq 0 \tag{13}$$

which implies that $x_1^{*(1)}$ and $x_1^{*(2)}$ have the same stability whenever $0 \leq x_1^{*(3)} \leq 1$ and have different stabilities otherwise. From Eq. (12c), we see that $x_1^{*(3)}$ is stable when

$$\alpha_1 \lambda_2^{(1)} + \alpha_2 \lambda_2^{(2)} > 0 \ , \tag{14}$$

which in turn implies that $x_1^{*(3)}$ has a different stability than $x_1^{*(1)}$ and $x_1^{*(2)}$ whenever $x_1^{*(3)} \in [0, 1]$.

III. SOLUTION FOR $|\mathcal{G}| = 2$ WITH STRATEGIC TRADEOFF

To emulate limited resources, we constrain the matrix $\boldsymbol{p}$ to the following structure

$$\boldsymbol{p} = \begin{pmatrix} p_1 & 1-p_1 \\ 1-p_2 & p_2 \end{pmatrix} \ , \tag{15}$$

where $0 \leq p_1, p_2 \leq 1$ are two free parameters. Combining Eq. (15) and the conservation condition $x_2 = 1 - x_1$, we obtain that the differential equation describing the dynamics of the $|\mathcal{G}| = 2$ scenario is

$$\dot{x}_1 = \frac{x_1(1-x_1)(1-p_1)}{x_1(1-p_1) + (1-x_1)p_2} - \frac{x_1(1-x_1)(1-p_2)}{x_1 p_1 + (1-x_1)(1-p_2)} \ , \tag{16}$$

From the complete analysis of this model given in Sec. II, we readily identify the three fixed points

$$x_1^{*(1)} = 0 \ , \tag{17a}$$
$$x_1^{*(2)} = 1 \ , \tag{17b}$$
$$x_1^{*(3)} = \left(1 + \frac{1-p_1}{1-p_2}\right)^{-1} \ . \tag{17c}$$

and draw the following conclusions:

- If $p_1 + p_2 < 1$, both $x_1^{*(1)}$ and $x_1^{*(2)}$ are unstable and $x_1^{*(3)}$ is stable:
  - Competitor 1 wins majority if $p_1 > p_2$;
  - It is a tie if $p_1 = p_2$;
  - Competitor 2 wins majority if $p_1 < p_2$.
- If $p_1 + p_2 = 1$, the system is static—$x_1(t) = x_1(0)$ for all time t—and the winning strategy is trivially determined by the initial conditions:
  - Competitor 1 wins majority if $x_1(0) > 1/2$;
  - It is a draw if $x_1(0) = 1/2$;
  - Competitor 2 wins majority if $x_1(0) < 1/2$.
- If $p_1 + p_2 > 1$, both $x_1^{*(1)}$ and $x_1^{*(2)}$ are stable and $x_1^{*(3)}$ is unstable:
  - Competitor 1 wins by unanimity if $x_1(0) > \left(1 + \frac{1-p_1}{1-p_2}\right)^{-1}$;
  - No draw is possible;
  - Competitor 2 wins by unanimity if $x_1(0) < \left(1 + \frac{1-p_1}{1-p_2}\right)^{-1}$.

IV. ANALYSIS OF FIXED POINTS IN THE CASE $|\mathcal{G}| > 2$

We change our focus to a scenario where more than two competitors compete to rally a majority of nodes. Eqs. (5) are non-linear, and their fixed points could be non-trivial in principle. However, from Eq. (2) we see that setting $x_i = 0$ constrains the phase space of the dynamics to the set of the remaining $\mathcal{G}\backslash i$ competitors. In other words, all pairwise fixed points (i.e., fixed points with at most two non-zero x_k^*) are already known. With $|\mathcal{G}| = 3$, the missing fixed points thus correspond to steady states where $x_k^* > 0 \ \forall \ k \in 1, 2, 3$; and so on as we add competitors. It turns out that (a) we can find the *unique* bulk fixed point of the $|\mathcal{G}|$ competitors case and (b) build the other fixed points recursively from the $|\mathcal{G}| - 1$ competitors cases, all the way down to $|\mathcal{G}| = 2$. For the sake of clarity, we define $g = |\mathcal{G}|$ in what follows.

A. Bulk fixed point

We define *bulk* fixed points as vectors $\vec{x}^*$ whose elements $x_k^* > 0 \ \forall \ k$, and for which $\dot{x}_k\big|_{\vec{x}^*} = 0 \ \forall \ k$. In this section, we obtain an analytical expression for the bulk fixed points and show that only a *single* one exists for each $g = 2, 3, ...$

By definition of the bulk fixed point, one must exclude $x_k^* = 0$ from $\vec{x}^*$, for all k. This is done by factoring out the leading x_k (i.e., the $x_k^* = 0$ root) from Eq. (5). We are left with the simpler system

$$\dot{x}_k = \sum_j p_{kj} x_j^* \varphi_j^* - 1 = 0 \qquad k = 1, 2, .., g \ . \tag{18}$$

Notice that Eq. (18) can be written in matrix form as

$$\boldsymbol{P}\boldsymbol{z} = \boldsymbol{1} \tag{19}$$

where $\boldsymbol{P}$ is the $g \times g$ matrix of parameters $\{p_{ij}\}$ and where the kth element of $\boldsymbol{z}$ is defined as $z_k := x_k^* \phi_k^*$. Provided that $\boldsymbol{P}$ is invertible, one finds

$$\boldsymbol{z} = \boldsymbol{P}^{-1}\boldsymbol{1} \quad \Longleftrightarrow \quad x_k^* \phi_k^* = s_k \qquad k = 1, 2, .., g \ , \tag{20}$$

where s_k is defined as the sum of the kth row of the invert of $\boldsymbol{P}$. It then becomes apparent that we have removed the non-linear terms of Eq. (2); multiplying by $(\varphi_k^*)^{-1}$ on both sides yields a system of linear equations for $\boldsymbol{x}^*$:

$$x_k^* - s_k \left(\sum_j p_{jk} x_j^* \right) = 0 \qquad k = 1, 2, .., g \ . \tag{21}$$

Note that in solving (21), we have no guarantee that $\sum_j x_j^* = 1$. It could therefore be that $\boldsymbol{x}^*$ is not a distribution. This problem is addressed by removing a redundant equation for x_g^* (without loss of generality), and using the normalization condition

$$\sum_{j=1}^{g} x_j^* = 1 \quad \Longleftrightarrow \quad x_g^* \equiv 1 - \sum_{j<g} x_j^* \ . \tag{22}$$

We are then guaranteed that the solutions of the resulting equation,

$$x_k^* - s_k \sum_{j<g} (p_{jk} - p_{gk}) x_j^* = s_k p_{gk} \qquad k = 1, 2, .., g-1 \ , \tag{23}$$

are *normalized* bulk fixed point. Eq. (22) can be used to recover x_g^* explicitly. The solution is unique as long as the system (23) is independent. The solution *can* be a non-bulk fixed point if the point is degenerated. Without loss of generality, suppose $x_{g-1}^* = 0$, such that the g-competitors "bulk-point" actually is not non-zero for all k. Equation (23) tells us that this can only happen if

$$s_{g-1} p_{g,g-1} = 0 \ , \tag{24}$$

i.e. if the sum of the $(g-1)^{\text{th}}$ row of the $\boldsymbol{P}^{-1}$ is zero. The other possibility, $p_{g,g-1} = 0$, can be avoided by removing a different row in Eq. 22, and will only lead to unavoidable degenerated bulk point in the ℓth dimension if $p_{j,\ell} = 0 \ \forall \ j$. Note that solutions *can* still reside outside of the simplex, i.e. $x_j^* < 0$ for some j, which we ignore as they are non-physical fixed points.

B. On fixed points recurrence

Let us consider, without loss of generality, that $x_g^* = 0$ and $\sum_{j<g} x_j^* = 1$. Then Eqs. (18) become

$$\dot{x}_k\Big|_{\vec{x}^*} = x_k^*\left[\sum_{j<g} x_j^* p_{kj}\varphi_j - 1\right] \qquad k = 1, 2, .., g-1\ , \tag{25a}$$

$$\dot{x}_g\Big|_{\vec{x}^*} = 0\ , \tag{25b}$$

$$\varphi_j\Big|_{\vec{x}^*} := \left[\sum_{\ell<g} x_\ell^* p_{\ell j}\right]^{-1} . \tag{25c}$$

where $\boldsymbol{x}^*$ is here any fixed point which satisfies the criterion stated above. We immediately recover Eqs. (18) for $g-1$ competitors by removing the trivial Eq. (25b), and redefining the summation limits. Therefore, given a g competitors system, one can obtain all fixed points where one of the components is null from the $g-1$ competitors system, recursively, all the way down to $g = 2$ which we fully solved previously.

To enumerate all fixed points, we therefore only need to

1. Find the bulk point for g competitors.
2. For all $\ell = 1, 2, ..., g$, set $x_\ell^* = 0$ and go back to step 1 with $g' = g - 1$.

The procedures stops once we reach $g' = 2$.

C. Scenario with strategic tradeoffs

In the scenario with strategic tradeoff, we have

$$p_{rs} = p_r\delta_{rs} + (1-p_r)\bar{\delta}_{rs} \equiv (1-p_r) + \delta_{rs}(2p_r - 1)\ . \tag{26}$$

The resulting $\boldsymbol{P}$ matrices are full rank matrices whenever there is at most one $p_r = 1/2$; all rows are independent from one another because of their diagonal element. Incidentally, any pair of competitors with both $p_r = 1/2$ will remain at their initial conditions as in the case with two competitors. In that case, we do not expect a single bulk point, but a *hyper-plane* of fixed points that connects $g-2$ edges parallel to the competitors pair edge, along every faces of the simplex (see Fig. 1 for example).

For all other cases, the inverse of $\boldsymbol{P}$ can be calculated exactly using the Sherman-Morrison formula and the matrix representation $\boldsymbol{P} = [2\,\mathrm{diag}(\boldsymbol{P}) - \boldsymbol{I}] + (\mathbf{1} + \boldsymbol{P})\mathbf{1}^T$, where $\boldsymbol{P}$ is the column matrix of probabilities p_r and $\mathbf{1}$ is a column matrix of 1. One finds

$$[\boldsymbol{P}^{-1}]_{rs} = \frac{\delta_{rs}}{2p_r - 1} - \frac{(1-p_r)}{(2p_r-1)(2p_s-1)}\left[1 + \sum_j \frac{1-p_j}{2p_j - 1}\right]^{-1} \tag{27}$$

Combining the recursive strategy of § IV B and Eq. (23) yields the position of all fixed points. Moreover, Eq. (27) allows us to compute s_k—we simply sum over each row of the inverse matrix—and obtain a condition for $s_k = 0$, i.e., a condition that predicts whether the "fixed bulk point" is degenerated and actually lives on the edges of the g-simplex (see Eq. 24). Doing so, we obtain the following condition for the non-existence of bulk point in the g competitors case (with strategic tradeoffs)

$$\sum_j \frac{p_k}{2p_j - 1} = \sum_j \frac{p_j}{2p_j - 1} \quad \text{for any } k = 1, .., g. \tag{28}$$

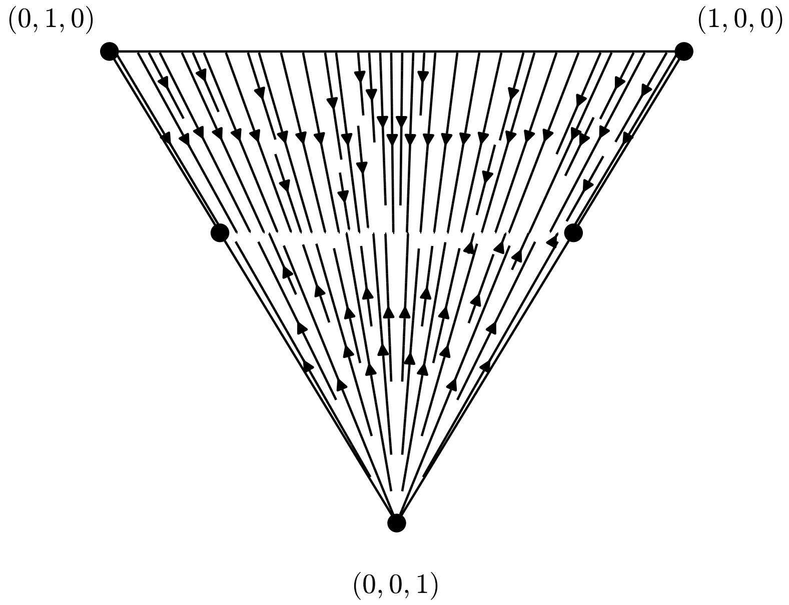

FIG. 1. **Example of hyperplanes in the phase space.** Competitors 1 and 2 (right and left corners respectively) are given the strategy $p = 1/2$, and the strategy of competitor 3 (bottom corner) is set to $p = 1/5$. The matrix $\boldsymbol{P}$ is not full rank, and the flow is therefore degenerated—the only interactions arise from the presence of competitor 3. A line of fixed points appears parallel to the (1,0,0)–(0,1,0) edge of the simplex.

V. COMPLETE TWITTER ANALYSIS AND MODELING

A. Raw data from Ref. [13] in main text.

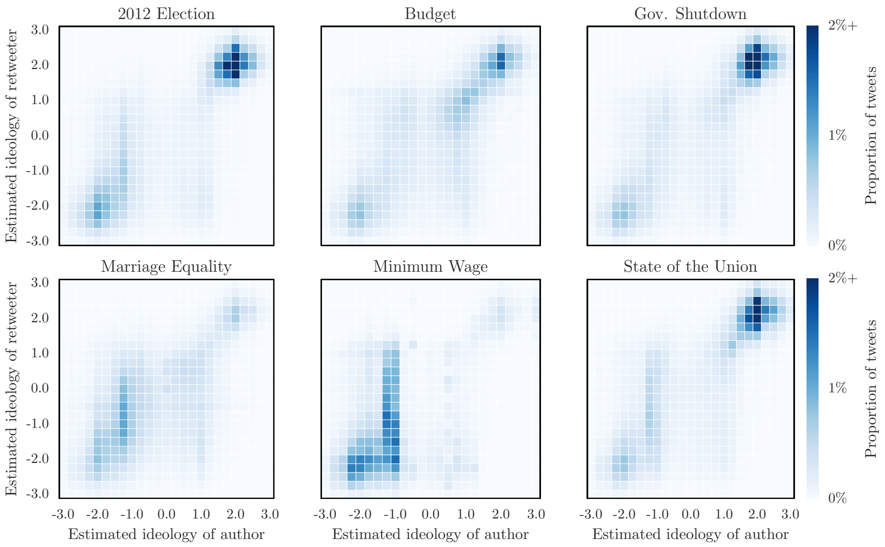

FIG. 2. **Political tweets**. Proportion of tweets by authors of estimated ideology x retweeted by users of estimated ideology y, where $x, y \in [-3, 3]$ denotes the estimated ideology of the users. Strongly liberal users are given a score of -3 and strongly conservative users are given a score of $+3$. Topics are, from left to right and top to bottom: 2012 election, federal budget, government shutdown, marriage equality, minimum wage and the state of the union.

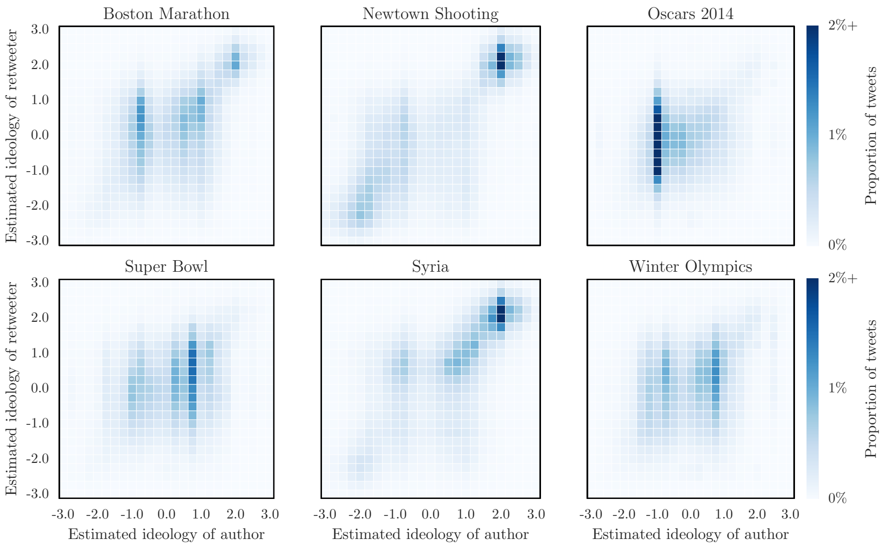

FIG. 3. **Non-political tweets**. Proportion of tweets by authors of estimated ideology x retweeted by users of estimated ideology y, where $x, y \in [-3, 3]$ denotes the estimated ideology of the users. Strongly liberal users are given a score of -3 and strongly conservative users are given a score of $+3$. Topics are, from left to right and top to bottom: Boston marathon, Newtown shooting, 2014 Oscars, Super Bowl, Syria, and the 2014 Winter Olympics.

B. Normalized matrices and coarse graining

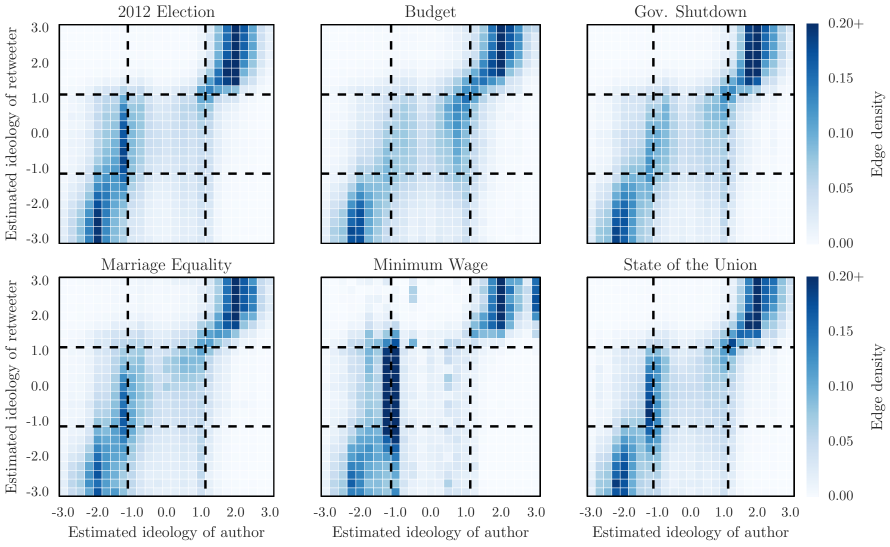

FIG. 4. **Strategies from political tweets.** Row-normalized retweet matrices yield potential strategy matrices. Dotted lines indicate the coarse-graining used to obtain a 3 competitor dynamics. Topics are, from left to right and top to bottom: 2012 election, federal budget, government shutdown, marriage equality, minimum wage and the state of the union.

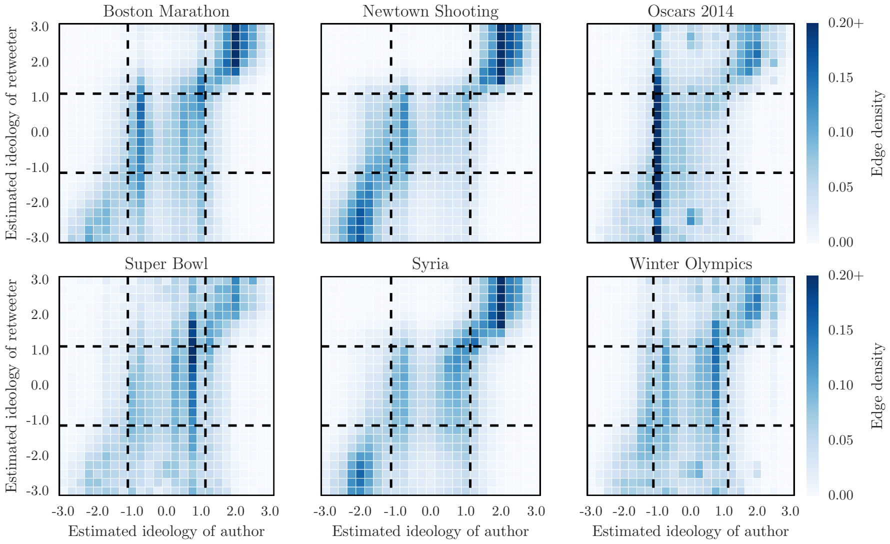

FIG. 5. **Strategies from non-political tweets.** Row-normalized retweet matrices yield potential strategy matrices. Dotted lines indicate the coarse-graining used to obtain a 3 competitor dynamics. Topics are, from left to right and top to bottom: Boston marathon, Newtown shooting, 2014 Oscars, Super Bowl, Syria, and the 2014 Winter Olympics.

C. Flows in the prevalence space

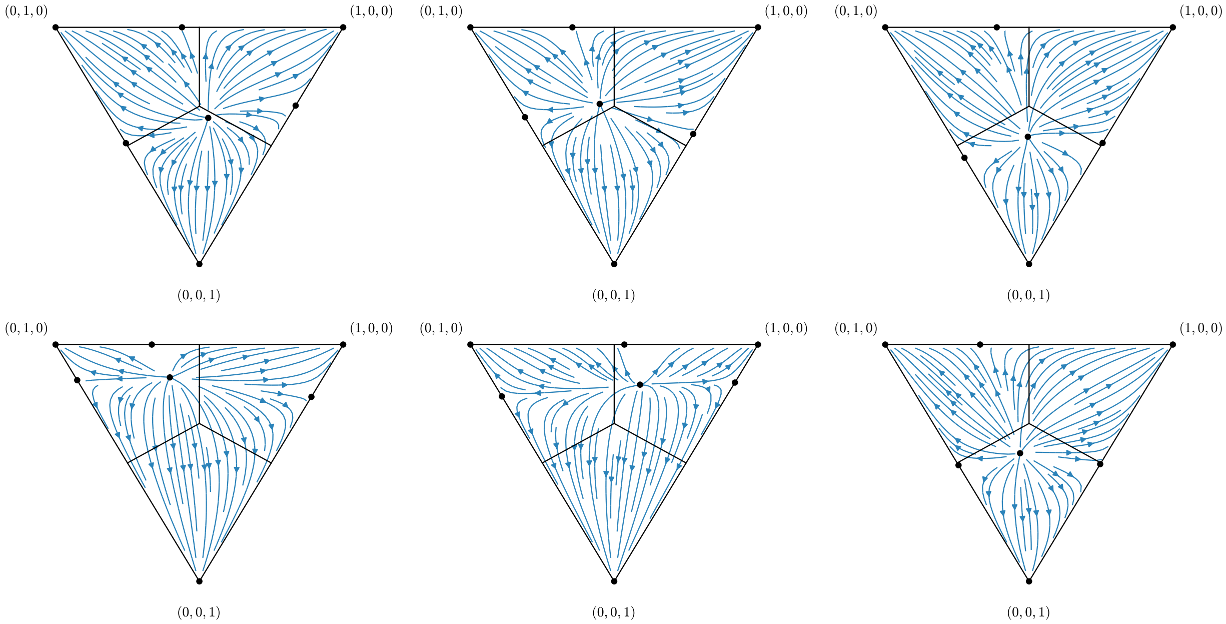

FIG. 6. **Flows in the prevalence space of political tweets.** The flows are obtained with our equations for arbitrary density matrices $\boldsymbol{P}$. Topics are, from left to right and top to bottom: 2012 election, federal budget, government shutdown, marriage equality, minimum wage and the state of the union. Point (1,0,0) correspond to a liberal outcome, (0,1,0) to a centrist outcome and (0,0,1) to a conservative outcome. All fixed points are shown with filled black circles, and their stability can be inferred from the trajectories around them.

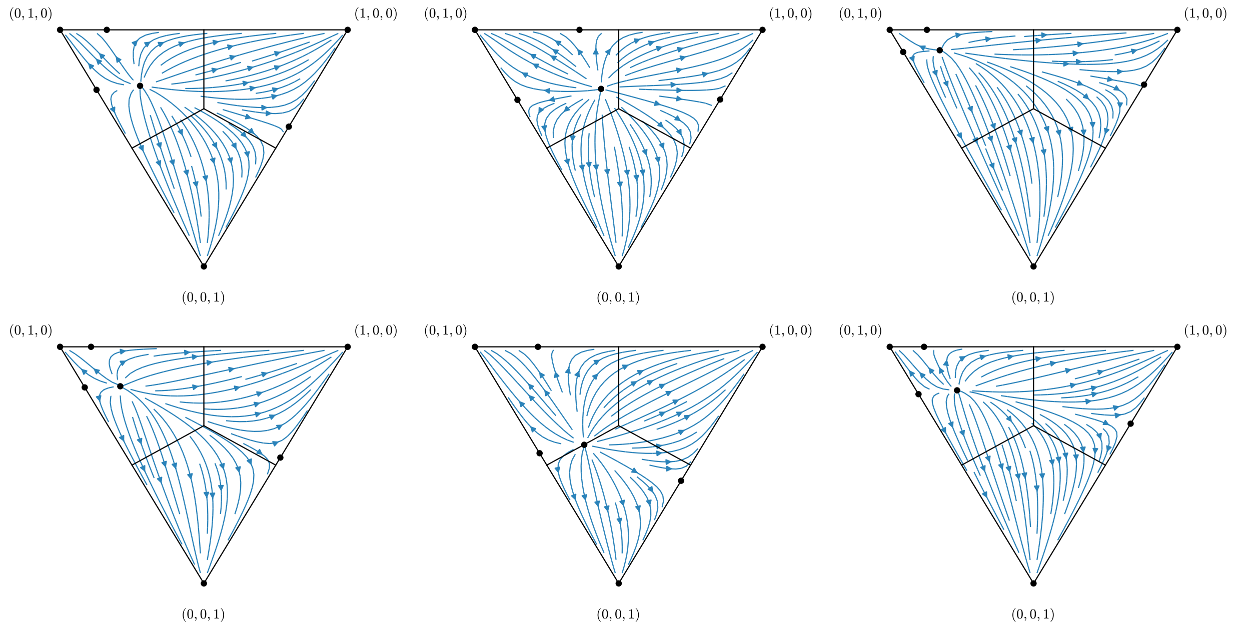

FIG. 7. **Flows in the prevalence space of non-political tweets.** The flows are obtained with our equations for arbitrary density matrices $\boldsymbol{P}$. Topics are, from left to right and top to bottom: Boston marathon, Newtown shooting, 2014 Oscars, Super Bowl, Syria, and the 2014 Winter Olympics. Point (1,0,0) correspond to a liberal outcome, (0,1,0) to a centrist outcome and (0,0,1) to a conservative outcome. All fixed points are shown with filled black circles, and their stability can be inferred from the trajectories around them.
